# The Endophytic Strain ZS-3 Enhances Salt Tolerance in *Arabidopsis thaliana* by Regulating Photosynthesis, Osmotic Stress, and Ion Homeostasis and Inducing Systemic Tolerance

**DOI:** 10.3389/fpls.2022.820837

**Published:** 2022-03-21

**Authors:** Li-Na Shi, Lan-Xiang Lu, Jian-Ren Ye, Hui-Min Shi

**Affiliations:** ^1^Co-Innovation Center for Sustainable Forestry in Southern China, College of Forestry, Nanjing Forestry University, Nanjing, China; ^2^Jiangsu Key Laboratory for Prevention and Management of Invasive Species, Nanjing Forestry University, Nanjing, China

**Keywords:** salt stress, *Bacillus megaterium*, salicylic acid, PGPR, ion regulation

## Abstract

Soil salinity is one of the main factors limiting agricultural development worldwide and has an adverse effect on plant growth and yield. To date, plant growth-promoting rhizobacteria (PGPR) are considered to be one of the most promising eco-friendly strategies for improving saline soils. The bacterium *Bacillus megaterium* ZS-3 is an excellent PGPR strain that induces growth promotion as well as biotic stress resistance and tolerance to abiotic stress in a broad range of host plants. In this study, the potential mechanisms of protection against salinity stress by *B. megaterium* ZS-3 in *Arabidopsis thaliana* were explored. Regulation by ZS-3 improved growth in *A*. *thaliana* under severe saline conditions. The results showed that ZS-3 treatment significantly increased the biomass, chlorophyll content and carotenoid content of *A*. *thaliana*. Compared to the control, the leaf area and total fresh weight of plants inoculated with ZS-3 increased by 245% and 271%, respectively; the chlorophyll a, chlorophyll b, and carotenoid contents increased by 335%, 146%, and 372%, respectively, under salt stress. Physiological and biochemical tests showed that ZS-3 regulated the content of osmotic substances in plants under salt stress. Compared to the control, the soluble sugar content of the ZS-3-treated group was significantly increased by 288%, while the proline content was significantly reduced by 41.43%. Quantification of Na^+^ and K^+^ contents showed that ZS-3 treatment significantly reduced Na^+^ accumulation and increased the K^+^/Na^+^ ratio in plants. ZS-3 also isolated Na^+^ in vesicles by upregulating *NHX1* and *AVP1* expression while limiting Na^+^ uptake by downregulating *HKT1*, which protected against Na^+^ toxicity. Higher levels of peroxidase and catalase activity and reduced glutathione were detected in plants inoculated with ZS-3 compared to those in uninoculated plants. In addition, it was revealed that ZS-3 activates salicylic acid (*NPR1* and *PR1*) and jasmonic acid/ethylene (*AOS*, *LOX2*, *PDF1.2*, and *ERF1*) signaling pathways to induce systemic tolerance, thereby inducing salt tolerance in plants. In conclusion, the results of this study indicate that ZS-3 has the potential to act as an environmentally friendly salt tolerance inducer that can promote plant growth in salt-stressed environments.

## Introduction

Soil salinity not only threatens plant growth and yield but also leads to a dramatic reduction in soil quality and availability (Abbas et al., 2019). It is a phenomenon of soil degradation caused by the enrichment of salt ions in the soil due to a combination of natural factors and human activities (Abbas et al., 2019). Low rainfall, high evaporation, native rocks, saline irrigation water, and poor water management are increasingly causing salinization problems in agricultural areas (Singh, 2021; Kumawat et al., 2022). In saline areas, the natural growth and development as well as the physiological metabolism of plants are severely disturbed by saline stress (Liu et al., 2020). The early response of salt stress is osmotic or drought stress, which can be sensed by cells within 5 min (Zelm et al., 2020). Ionic stress is induced at a later stage, when Na^+^ accumulation in the soil not only rapidly reduces water availability but also slowly accumulates in the shoots (Zelm et al., 2020). In addition, plants produce excess reactive oxygen species (ROS), which interfere with the electron transport chain in chloroplasts and mitochondria (Gill and Tuteja, 2010). Therefore, a range of regulatory mechanisms have evolved in response to adversity stress. For example, low molecular weight organic compounds such as amino acids, sugars, inorganic ions, and organic acids accumulate in the cytoplasm to reduce the osmotic potential (Abbas et al., 2019). To protect themselves from Na^+^ toxicity, plants enhance Na^+^ tolerance by regulating Na^+^ transport, translocation and intracellular compartmentalization, which are mediated by ion transport-associated proteins such as high-affinity K^+^ transporter 1 (*HKT1*) and Na^+^/H^+^ reverse transporter proteins (*NHXs*; Qin et al., 2016). However, defense mechanisms are not widespread in all plants. Sustainable methods to improve salt tolerance in saline soil crops are urgently needed.

Various strategies, such as improved irrigation techniques, physical flushing, leaching and scraping of saline soils, traditional genetic breeding, and genetic engineering of plants, have been used to mitigate this problem (Abbas et al., 2019; Kumar Arora et al., 2020). However, there are limitations to these methods, and some approaches may even have additional negative impacts on ecosystems. For example, irrigation techniques are usually very expensive and unsustainable (Singh, 2021). Soil leaching requires relatively low soil moisture and a low water table, making this a difficult approach for agro-ecosystems, and important nutrients in the soil are lost during the leaching process (Kumar Arora et al., 2020). Traditional genetic breeding and plant genetic engineering to produce salt-tolerant crops are widely considered to be effective strategies, but these methods usually require a lengthy selection and validation process (Kumawat et al., 2022). Moreover, it is still uncertain whether transgenic crops will be universally accepted (Qin et al., 2016). To date, plant growth-promoting rhizobacteria (PGPR) are considered to be one of the most promising eco-friendly strategies for improving saline soils (Kumar Arora et al., 2020).

PGPR, as biological inoculants, usually have various direct mechanisms, such as nitrogen fixation, IAA production and phosphorus solubilization, to promote plant growth. PGPR also have indirect mechanisms of plant growth promotion such as siderophore production, volatile organic compounds (VOC), and ACC deaminase (Etesami and Alikhani, 2016; Gong et al., 2020; Kumar Arora et al., 2020). When applied in the field, PGPR resulted in an improvement of the texture, EC, pH, and organic matter of saline lands (Kumar Arora et al., 2020). There is clear evidence that PGPR are essential for promoting plant adaptation to salinity (Qin et al., 2016; Kumar Arora et al., 2020). PGPR contribute to the tolerance of many plants to abiotic stresses by synthesizing osmotic substances, balancing ionic homeostasis in plants and inducing antioxidant mechanisms (Lucke et al., 2020a). *Bacillus amyloliquefaciens* FZB42 VOCs alleviate Na^+^ toxicity by significantly reducing Na^+^ contents in whole plants and upregulating the expression of ion transport genes (Liu et al., 2020). Increased accumulation of osmoregulatory substances, such as proline and soluble sugars, has been reported to be an important mechanism by which PGPR promote plant growth under saline conditions. *Kocuria rhizophila* Y1 significantly increased maize biomass, and an elevated proline content, soluble sugar content and K^+^/Na^+^ ratio were also observed under NaCl treatment (Li et al., 2020a). Shahid et al. (2021) also indicated that PGPR, such as the salinity-tolerant *Kosakonia sacchari* MSK1, increased the chlorophyll content and carotenoid content of *Vigna radiata* under different levels of salinity, mitigating the negative effects of salt stress on plant photosynthesis.

Molecular studies of gene expression, protein synthesis, and metabolite production in plants also confirm that the PGPR signaling network confers tolerance to plant stress (Ilangumaran and Smith, 2017). Jasmonic acid (JA), ethylene (ET), and salicylic acid (SA) are the major signaling molecules in plant stress responses, and their signaling cascades have been extensively studied (Khan et al., 2020). JA is a positive regulator of salt tolerance, which together with ethylene (ET) induces defense responses to abiotic stresses in plants (Takeuchi et al., 2011; Liu et al., 2017). SA plays an important role in systemic acquired resistance (SAR) to attack by biotic pathogens (Niu et al., 2016; Timmermann et al., 2017). These two processes show an antagonistic relationship when responding to biotic stresses (Timmermann et al., 2017). Hormone metabolism in signaling networks mediated by PGPR provides cross-protective properties and attenuates abiotic stress (Khan et al., 2020). *Bacillus amyloliquefaciens* FZB42 confers salt tolerance by upregulating the plant JA/ET pathway *via* PGPR-plant interactions (Liu et al., 2017). However, to our knowledge, only a few studies have revealed that PGPR induce salt tolerance in plants *via* SA (Liu et al., 2017). Furthermore, there is a general consensus that crosstalk between JA and SA is widely present in biotic stresses but is rarely reported under salt stress.

Previous studies have shown that *Bacillus megaterium* ZS-3 (ZS-3) is an excellent PGPR with the ability to solubilize phosphorus and produce organic acids and IAA (Lu et al., 2018; Qian and Ye, 2020). It can induce plant growth and coping responses to biotic stresses. We hypothesize that ZS-3 effectively mitigates the negative effects of salt stress on plants and reveal the physiological and molecular mechanisms of ZS-3-induced salt stress tolerance in plants so that this strain can be more scientifically applied as a biological inoculant to improve the productivity of crops in saline soils. In this work, the effects of ZS-3 on plant morphological and biochemical properties, such as plant growth, photosynthetic capacity, Osmotic substance accumulation (proline and soluble sugars), ROS production, antioxidant enzyme activity, and ion uptake (K ^+^ and Na ^+^) were evaluated in the presence and absence of salt stress. In addition, the transcript levels of genes related to plant growth and stress tolerance, such as Na^+^ extrusion, SA, JA and ET-mediated defense response genes, were also dynamically observed.

## Materials and Methods

### Plant Materials and Treatments

*Arabidopsis thaliana* ecotype Columbia-0 (Col-0) seeds were surface sterilized with NaOCl (10%, v/v) for 5 min, followed by three washes with 75% ethanol and further rinsing at least three times with sterilized water. They were then placed on 10-cm Petri dishes containing one-half-strength Murashige and Skoog ( $\frac{1}{2}$ MS) agar medium supplemented with 1% sucrose and 0.8% agar (pH 5.8). All plates were covered and sealed with Parafilm and were stored at 4°C in the dark for 48 h to ensure stratification. Seeds were grown for 6 days under long-day conditions (16 h light/8 h dark) in a light incubator maintained at 22 ± 2°C.

### Bacterial Cultures and Inoculation

The *B. megaterium* strain ZS-3 (GenBank: OM085668) was isolated from the branch tissue of *Cinnamomum camphora*. ZS-3 was grown on Luria-Bertani (LB) medium at 28°C for 16 h and then collected, washed, and diluted with phosphate buffered saline (PBS) solution to an OD_600_ of 0.1. Five microliters of bacterial dilution was inoculated 2 cm from the roots of 6-day-old Col-0 seedlings and placed vertically on $\frac{1}{2}\;$ MS plates.

### *In vitro* Salt Stress

Six-day-old Col-0 seedlings were placed on $\frac{1}{2}$ MS plates with an additional 0 m mol/L (mM) NaCl or 100 mM NaCl (high salt stress), for two treatments with ZS-3 and without ZS-3 inoculation. There were 20 replicate plates for each treatment and eight seedlings in each plate. Seedlings were harvested 14 days after inoculation for photography and analysis.

### Growth and Biomass Yield

After 14 days of cultivation, plants were detached from the $\frac{1}{2}$ MS plates for biomass analysis. The plant fresh weight was measured by an analytical scale. The lengths of the main and lateral roots of the plants were measured with a ruler. The total leaf surface area was quantified by a root system scanner (Expression 11000XL, Japan).

### Determination of Chlorophyll and Carotenoid Contents

The chlorophyll content of the leaves was measured as previously described Azarmi et al. (2015). Fresh leaves (0.1 g) were soaked and ground in 80% acetone. Absorbance was recorded at 663.6, 646.6, and 470 nm on a Multiskan spectrum (Thermo, United States) to quantify the chlorophyll a, chlorophyll b and carotenoids. Chlorophyll and carotenoid concentrations were calculated as follows:


$$Chl.a\left(\mu g/mLFW\right)=12.25\times OD_{663.6}-2.55\times OD_{646.6}$$

$$Chl.b\left(\mu g/mLFW\right)=20.31\times OD_{646.6}-4.91\times OD_{663.6}$$

$$T\mathrm{otal}\;\mathrm{car}.\left(\mu g/mLFW\right)=\frac{1000OD_{470}-3.27\times \left(Chl.a\right)-104\times \left(Chl.b\right)}{227}$$

### Determination of Osmoregulatory Substances

Proline and total soluble sugars were extracted from 0.1 g of fresh leaves. The proline content was determined using the colorimetric assay of Bates et al. (1973). The absorbance at 520 nm was determined with a spectrophotometer (Lambda 365, United States). A standard curve was made with L-proline, and distilled water was used as a control.

The procedure was used to assess the soluble sugar content (μg mg^−1^ FW). Serial dilutions of D-glucose were used to make the standard curve. The optical density of the mixture was measured at 620 nm with a spectrophotometer (Lambda 365, United States).

### Antioxidant Enzyme Activities and GSH, H_2_O_2,_ and MDA Contents

CAT, POD, GSH, H_2_O_2_, and MDA were extracted from 0.1 g of fresh leaves. All absorbance readings were taken with a Multiskan spectrum (Thermo, United States).

The catalase (CAT) activity (nmol min^−1^ g^−1^ FW) of whole plants was determined by a CAT kit (Comin, Suzhou, China). The absorbance was recorded at 240 nm (Rady et al., 2019).

The peroxidase (POD) activity (U g^−1^ FW) of whole plants was assayed by a POD kit (Comin, Suzhou, China). The absorbance was recorded at 470 nm.

The content (μmol g^−1^ FW) of reduced glutathione (GSH) was determined by a reduced GSH kit (Comin, Suzhou, China). The absorbance was recorded at 412 nm.

To determine the hydrogen peroxide (H_2_O_2_) content, whole plants were homogenized in an ice bath with acetone (1 ml). The resulting homogenate was centrifuged at 8,000 rpm for 10 min at 4°C. Determination was carried out according to the procedure of the H_2_O_2_ kit (Comin, Suzhou, China), and the absorbance was read at 415 nm.

The MDA content in leaf samples was determined by the thiobarbiturate reaction according to a previously described method (Kumar et al., 2013).

### Determination of K^+^, Na^+^, and K^+^/Na^+^ Contents

Fresh leaves and roots were harvested and dried at 85°C for 2 days to obtain a constant weight. The dried samples were ground with a mortar and filtered through a sieve with a pore size of 0.44 mm. The resulting powder (50 mg) was treated with a mixture of acids (5 ml HNO_3_ in 1 ml HClO_4_) at room temperature overnight. The sample was then digested at 200°C until the volume of the sample was reduced to 0.5 ml. Ion concentrations (K^+^ and Na^+^) were determined by flame spectrophotometry (AOE, China) based on the method described in Wolf (1982).

### Real-Time Quantitative PCR

Quantitative real-time PCR (qRT–PCR) was performed on uninoculated and ZS-3-inoculated Col-0 seedlings to quantify the expression levels of ion transporter genes (*NHX1*; Na^+^/H^+^ antiporter, *AVP1*; encodes vacuolar H^+^-pyrophosphatase and *HKT1*; high-affinity K^+^ transporter 1) as well as plant immunity-related genes, including markers of the SA signaling pathway (*PR1*; pathogenesis-associated 1, *NPR1* nonexpressor of pathogenesis-related 1) and the JA/ET-mediated defense response pathway (*PDF1.2*; plant defensin 1.2, *LOX2*; lipoxygenase 2, *AOS*; allene oxide synthase, *ERF1*; ethylene-responsive factor 1). Actin in Col-0 was used as an internal reference. The primers used in this study are listed in Supplementary Table S1.

RNA was extracted from the plants after 3, 5 and 8 days of salt treatment using the Fast Pure Plant Total RNA Isolation Kit (Vazyme, Nanjing) following the manufacturer’s instructions. Purified RNA was quantified using a UV scanning NanoDrop™ 2000 spectrophotometer (Thermo Scientific, Germany). Absorbance ratios at 260, 280 and 230 nm were recorded to assess the purity of the RNA. RNA was electrophoresed on a 1.5% (w/v) formaldehyde agarose gel and visualized using a UV transilluminator. The remaining genomic DNA was removed, and the first-strand cDNA was synthesized by HiScript II Q RT SuperMix for qPCR (Vazyme, Nanjing). qRT–PCR analysis was performed using SYBR qPCR Master Mix (Vazyme, Nanjing) with an ABI Prism 7,500 (Applied Biosystems, Foster City, CA, United States). PCR systems were set up as described in Li et al. (2020b). PCR amplification conditions were set up as described in Showmy and Yusuf (2020). For each treatment, three biological replicates were included. Gene expression levels were determined according to the 2^-ΔΔCt^ method (Fu et al., 2020).

### Data Analyses

The completely randomized design was the layout in this study. Statistical analysis of the data was performed using SPSS (version 22.0; SPSS, Inc.). One-way analysis of variance (ANOVA), Student’s *t*-test (*t*-test) and Duncan’s multiple range test were performed. Data represented in graphs are means ± standard deviations (SD) of at least three replicate samples (*n* = 3).

## Results

### ZS-3 Promotes Plant Growth and Increases Plant Salt Tolerance

**Figure 1:**
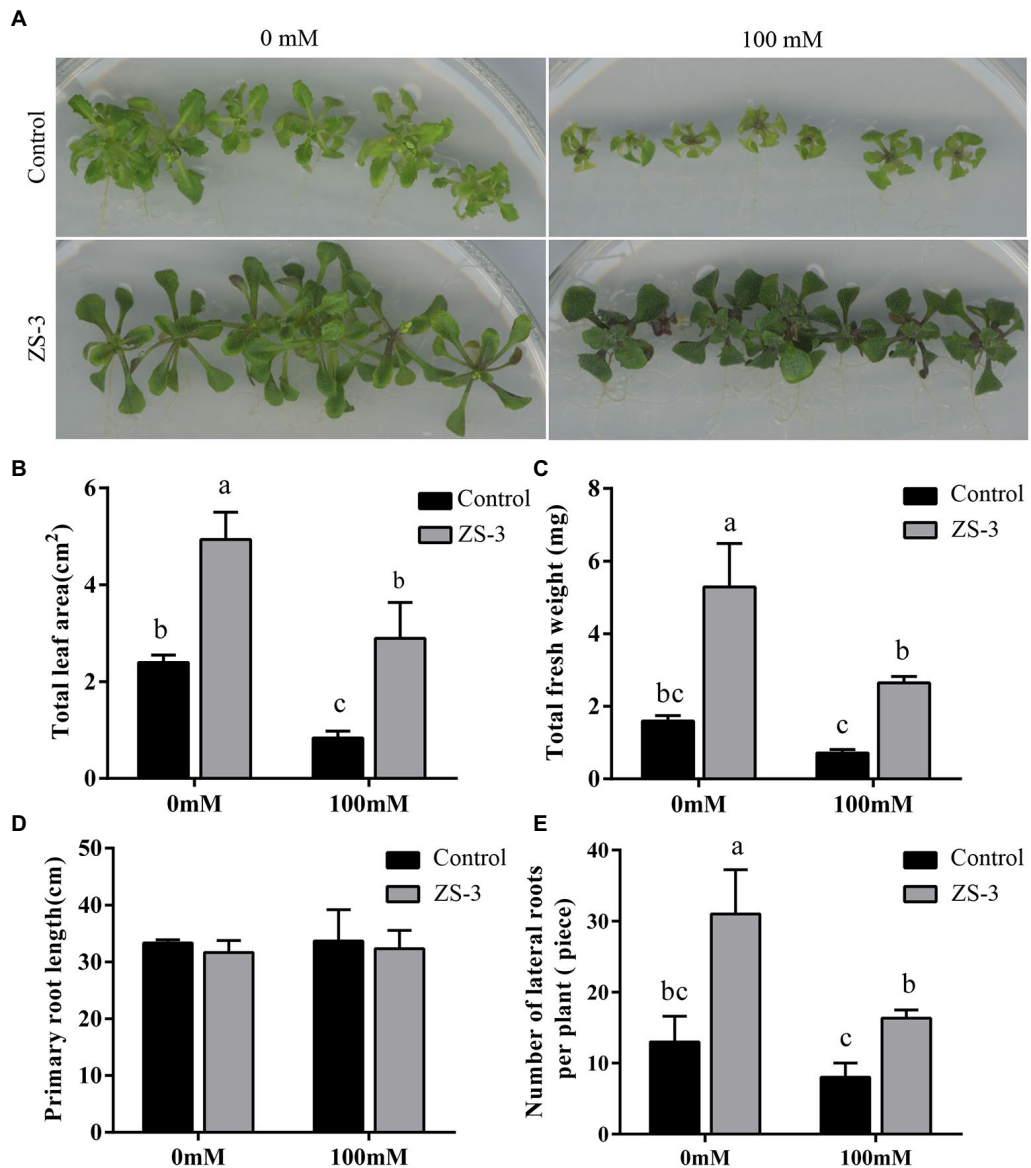
Inoculation with ZS-3 increases biomass in *Arabidopsis thaliana*. Plants were treated with salt stress for 14 days, pictures **(A)** were collected, and the plants were analyzed for total leaf area **(B)**, total fresh weight **(C)**, primary root length **(D)**, and lateral root number **(E)**. The results are the mean ± standard deviation from three independent experiments. Different lowercase letters above the bars represent significant differences based on one-way ANOVA (Duncan’s multiple range, *p* < 0.05).

To evaluate the effect of ZS-3 on the growth and salt tolerance of plants, *in vitro* salt stress experiments were carried out. After 14 days of coculture, plant biomass, including fresh weight, leaf area, and primary and lateral roots, was analyzed. *Arabidopsis thaliana* subjected to salt stress due to high concentrations of NaCl had a significantly smaller leaf area than that under the unstressed treatment, but ZS-3 treatment significantly increased the biomass of the plant and darkened the leaves (Figure 1A). In the absence of salt stress, ZS-3 treatment increased the leaf area of plants by 106% compared to that of uninoculated plants, while under salt stress, the leaf area of plants under the ZS-3 treatment increased by 245% compared to the control (Figure 1B). In addition, ZS-3 treatment increased the total fresh weight (FW) of plants by 231% and 271% in the absence and presence of salt stress, respectively (Figure 1C). The data showed that ZS-3 significantly increased the number of lateral roots of plants, while there was no significant difference in primary root length between inoculated and uninoculated plants (Figures 1D,E). These results indicated that ZS-3 promoted plant growth and enhanced plant tolerance to salt stress.

### Enhanced Biosynthesis of Photosynthetic Pigments

**Figure 2:**
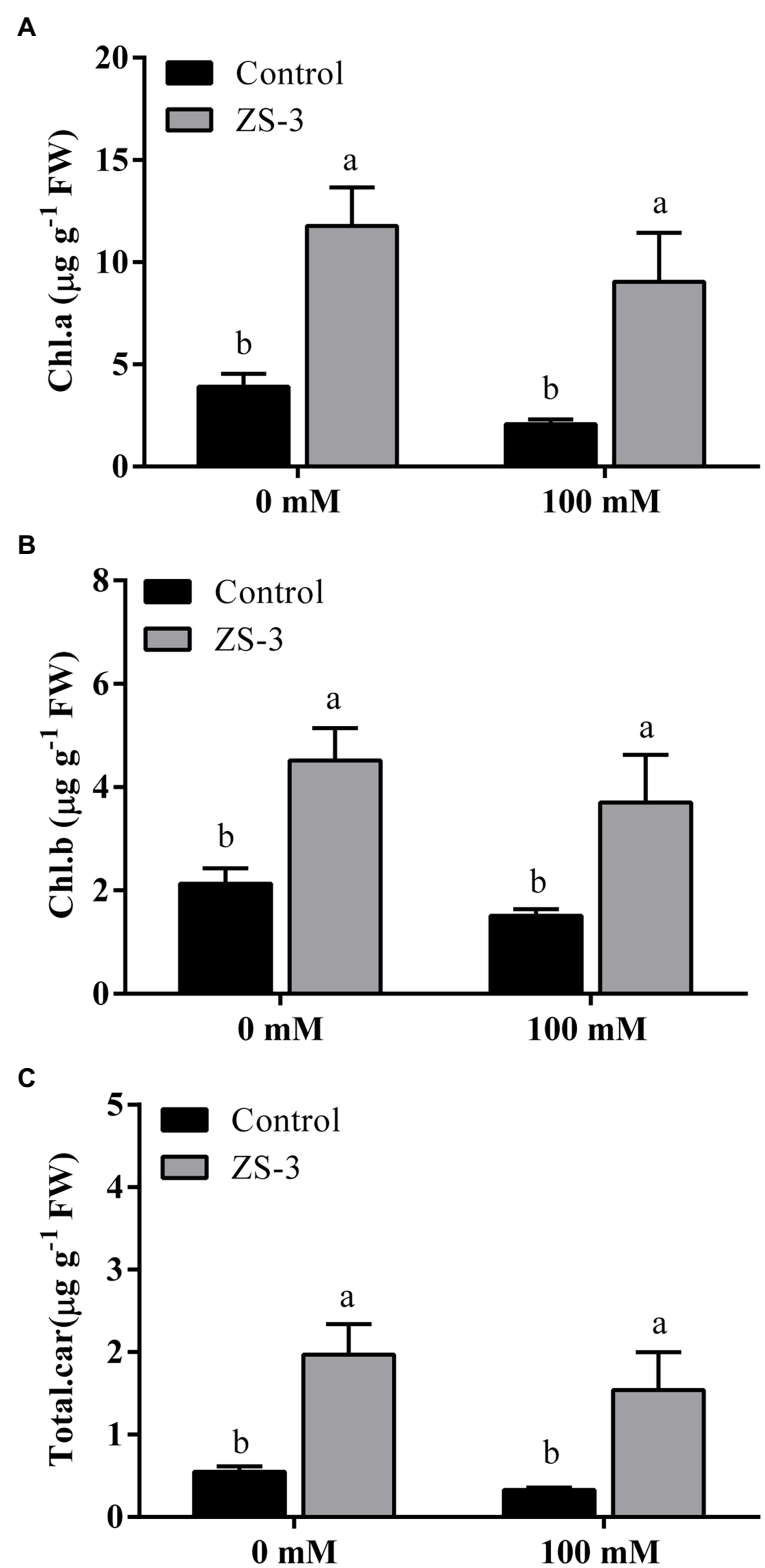
Effect of inoculation with ZS-3 on chlorophyll a **(A)**, chlorophyll b **(B)** and carotenoid **(C)** contents in *Arabidopsis thaliana*. The results are the mean ± standard deviation from three independent experiments. Different lowercase letters above the bars represent significant differences based on one-way ANOVA (Duncan’s multiple range, *p* < 0.05).

ZS-3 inoculation significantly induced the biosynthesis of pigments under either the presence or absence of salt stress (Figure 2). In the absence of salt stress, ZS-3 treatment increased chlorophyll a, chlorophyll b and carotenoids by 202%, 112%, and 260%, respectively, compared to levels in uninoculated plants. Under 100 mM salt stress, the ability of plants to produce chlorophyll and carotenoids was inhibited compared to the absence of salt stress. However, inoculation with ZS-3 increased the content of chlorophyll a, chlorophyll b and carotenoids by 335%, 146% and 372%, respectively. This suggests that ZS-3 increases the photosynthetic capacity of plants in the presence and absence of salt stress.

### Regulation of Osmoregulatory Substances by ZS-3

**Figure 3:**
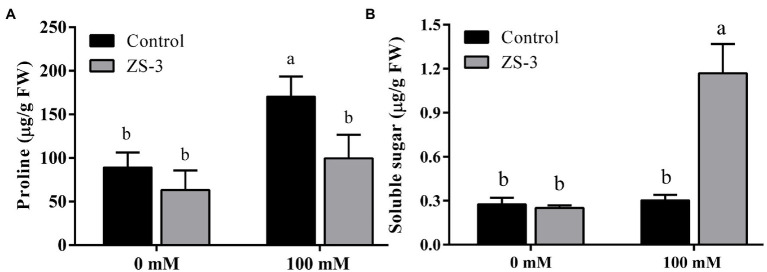
Effect of inoculation with ZS-3 on the proline content **(A)** and soluble sugar content **(B)** content of *Arabidopsis thaliana*. The results are the mean ± standard deviation from three independent experiments. Different lowercase letters above the bars represent significant differences based on one-way ANOVA (Duncan’s multiple range, *p* < 0.05).

The accumulation of compatible substances protects cells from damage caused by salt stress. Therefore, the contents of proline and soluble sugars in plant leaves were measured. In the absence of salt stress, there was no significant difference in plant proline (Figure 3A) and soluble sugar contents (Figure 3B) between the control and ZS-3-treated groups. The soluble sugar content (Figure 3B) was significantly increased by 288% and the proline content (Figure 3A) was reduced by 41.43% in plants inoculated with ZS-3 compared to the control levels under 100 mM salt stress conditions.

### ZS-3 Mediates Ionic Homeostasis in Plants

**Figure 4:**
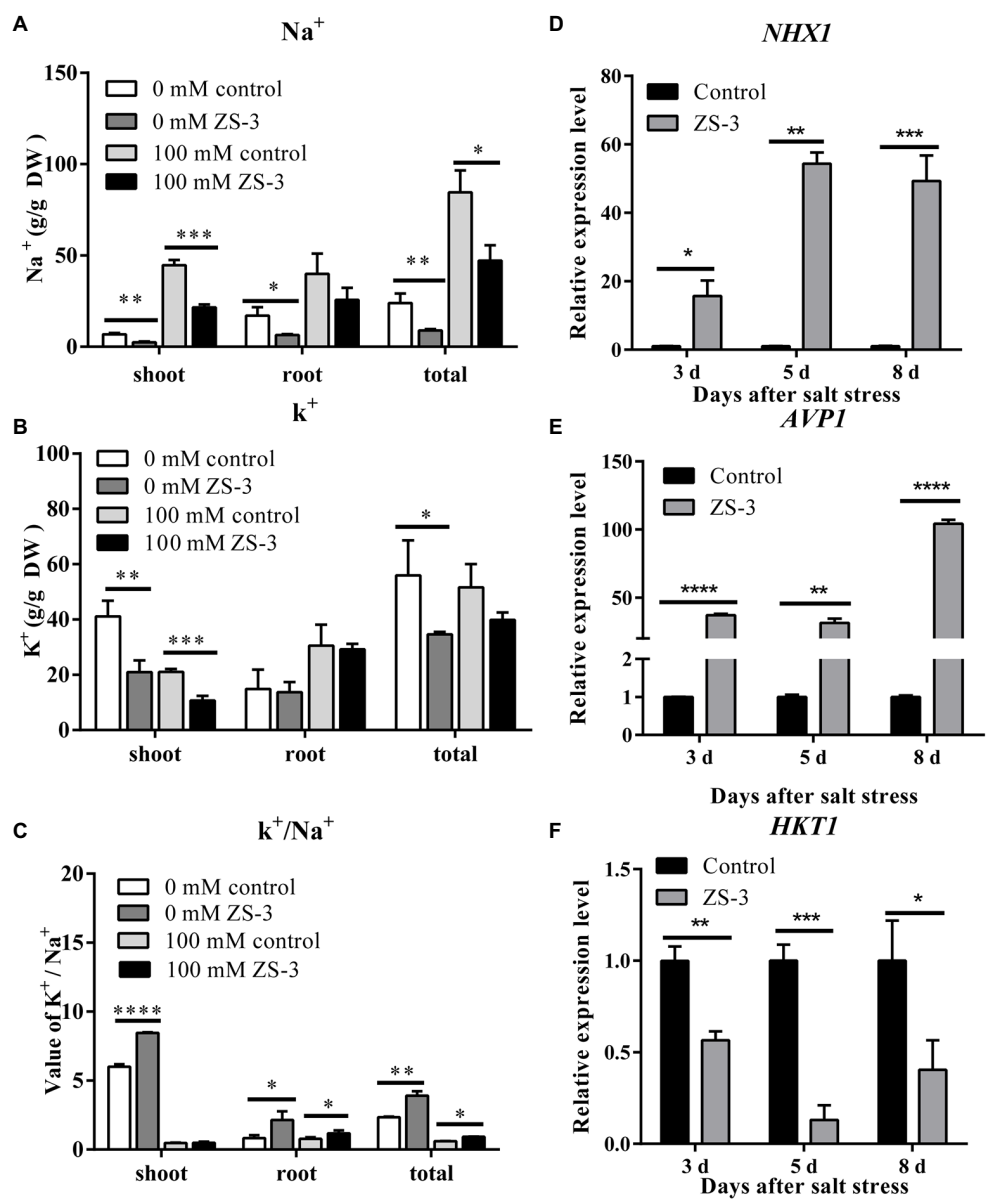
Effect of inoculation with ZS-3 on ion uptake in *Arabidopsis thaliana*. Na^+^ accumulation **(A)**, K^+^ production **(B)**, and the K^+^/Na^+^ ratio **(C)** in *Arabidopsis thaliana* roots and leaves were measured at 14 days after inoculation with strain ZS-3 in the absence or presence of 100 mM NaCl. The expression levels of the Arabidopsis thaliana defense-related genes *NHX1* **(D)**, *AVP1* **(E)** and *HKT1* **(F)** were quantified by qRT–PCR at days 3, 5 and 8 after inoculation with strain ZS-3. *Arabidopsis thaliana* inoculated with inactivated bacterial dilutions was used as a control. The results are the means ± standard deviations from three independent experiments. Asterisks indicate significant differences between treatments according to Student’s *t*-test (*p < 0.05; **p < 0.01; ***p < 0.001; ****p < 0.0001).

To reveal the effect of inoculation with strain ZS-3 on the major ions of Col-0 seedlings under NaCl stress, K^+^ and Na^+^ levels were measured. The salt stress treatment induced significantly higher Na^+^ content in the leaves, roots and whole plants than the treatment without salt stress (Figure 4A). Na^+^ accumulation in whole plants and leaves was reduced by 51.64% and 44.17%, respectively, in the ZS-3-treated group compared to the control, while the Na^+^ content in plant roots was not significantly affected. In the presence or absence of salt stress, ZS-3 significantly reduced the K ^+^ content of plant leaves compared to the uninoculated treatment (Figure 4B). In addition, the K^+^/Na^+^ ratio is considered an indicator of the balance between K^+^ and Na^+^ uptake by plants. In the absence of salt stress, ZS-3 significantly increased the K^+^/Na^+^ ratio in the shoots and roots of the plants. In the presence or absence of salt stress, inoculation with ZS-3 increased the K^+^/Na^+^ ratio of whole plants by 66.59% and 40.42%, respectively, compared to the control (Figure 4C).

Ion transporters are known as terminal determinants of ionic homeostasis under salt-stressed conditions. ZS-3 inoculation markedly enhanced the expression levels of the *NHX1* and *AVP1* genes after day three under salt stress conditions compared to those in the noninoculated groups (Figures 4D,E). However, in contrast to the other two genes, *HKT1* was negatively regulated in the ZS-3 treatment group on the 3rd day (Figure 4F).

### ZS-3 Increases the Antioxidant Content of Plants

**Figure 5:**
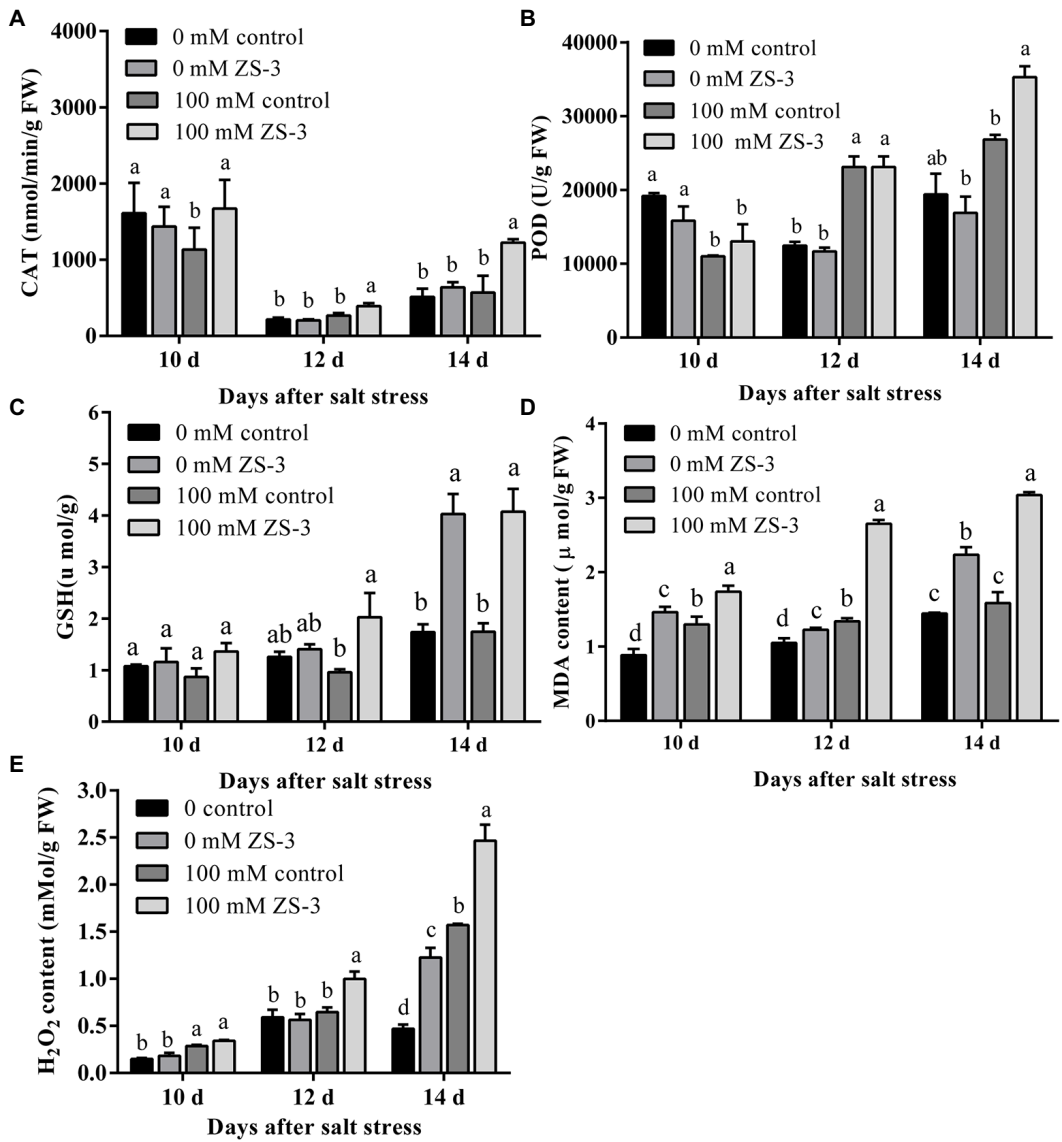
Effect of ZS-3 inoculation on antioxidant substances and ROS in *Arabidopsis thaliana*. CAT **(A)**, POD **(B)**, GSH **(C)**, MDA **(D)**, and H_2_O_2_ **(E)** were measured in *Arabidopsis thaliana* at 10, 12, and 14 days after inoculation with strain ZS-3 in the absence or presence of 100 mM NaCl. The results are the mean ± standard deviation of three independent experiments. Different lowercase letters on the bars represent significant differences between treatments based on one-way ANOVA (Duncan’s multiple range, *p* < 0.05).

Antioxidant enzyme activity and nonenzymatic antioxidant levels in leaves were quantified at 10, 12 and 14 days to investigate the potential role of ZS-3 in the presence or absence of salt stress. Under the absence of salt stress, inoculation with ZS-3 did not cause higher POD and CAT activity than that in uninoculated plants, but inoculation with ZS-3 significantly increased GSH levels at 14 days (Figure 5C). Under salt stress conditions, CAT, POD and GSH contents were significantly increased in ZS-3-inoculated plants. Compared to the uninoculated treatment, the CAT activity of the bacteria-treated plants showed a continuous upward trend, being significantly increased by 82.68%, 44.83%, and 77.19% at 10, 12, and 14 days, respectively (Figure 5A). POD activity increased significantly by 31.53% compared to those of the control at 14 days (Figure 5B). GSH contents increased by 111% and 133% at 12 and 14 days, respectively, compared to the control levels (Figure 5C).

However, in the presence or absence of salt stress, the contents of H_2_O_2_ and MDA were significantly increased in ZS-3-inoculated plants compared to those in noninoculated plants (Figures 5D,E). This would suggest that although ZS-3 enhanced plant antioxidant enzyme activity and nonenzymatic antioxidant levels, inoculation with the ZS-3 strain did not result in ROS scavenging and even exposed the plants to oxidative stress.

### ZS-3 Activates the Systemic Defense Response in Plants

**Figure 6:**
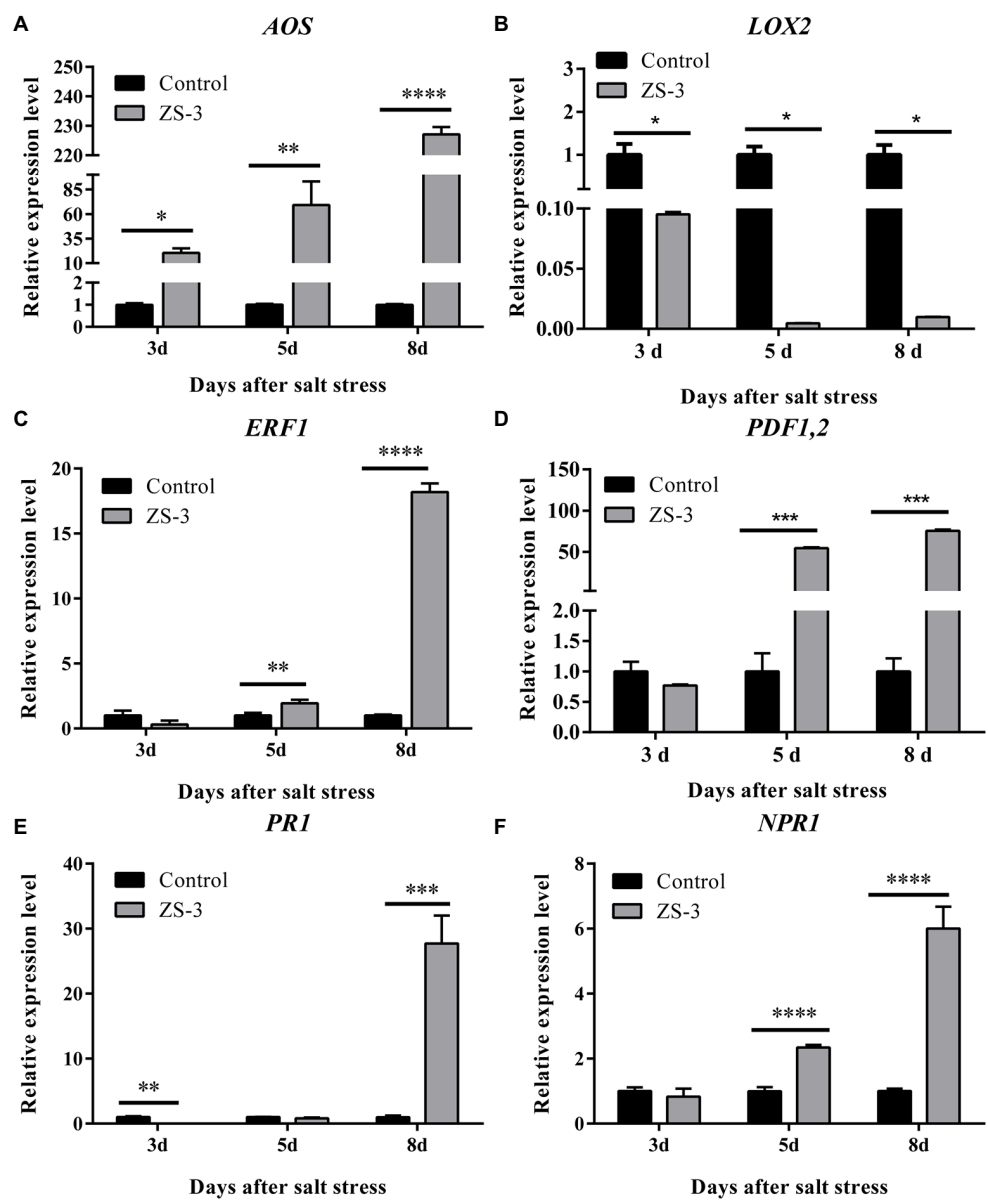
Real-time polymerase chain reaction (PCR) analysis of gene expression under salt stress. The expression levels of the *Arabidopsis thaliana* defense-related genes *AOS* **(A)**, *LOX2* **(B)**, *ERF1* **(C)**, *PDF1.2* **(D)**, *PR1* **(E)**, and *NPR1* **(F)** were quantified by qRT–PCR at days 3, 5 and 8 after inoculation with strain ZS-3. *Arabidopsis thaliana* inoculated with inactivated bacterial dilutions was used as a control. The results are the mean ± standard deviation of three independent experiments. Asterisks on the bars indicate significant differences between treatments based on *t*-test analysis (*p < 0.05; **p < 0.01; ***p < 0.001; ****p < 0.0001).

The expression of genes related to the SA and JA/ET pathways was examined after ZS-3 inoculation in *A*. *thaliana*, and the results showed that all three pathway-related genes examined were activated to varying degrees by the ZS-3 strain. In the JA pathway, *AOS*, a key gene for JA synthesis, showed an upward trend from days 3 to 8 (Figure 6A), while *LOX2* was always downregulated (Figure 6B). *ERF1*, a transcription factor downstream of the ET signaling pathway, was upregulated (2.0-fold) at day 5, with the highest (18.2-fold) expression at day 8 (Figure 6C). *PDF1.2*, a critical gene in the JA/ET pathway, was significantly upregulated (54.4-fold) from day 5 onward (Figure 6D). In the SA pathway, the defense protein *PR1* and the most critical receptor protein *NPR1*, which can be induced by SA, reached their highest expression simultaneously at day 8 (27.7-fold and 6.0-fold, respectively) (Figures 6E,F). Evidently, the SA and JA/ET pathways were all activated by strain ZS-3.

## Discussion

High salinity causes osmotic stress, ion toxicity, oxidative damage, and nutrient imbalance in plants, which reduces photosynthetic rates, inhibits plant growth, and reduces plant yield and quality. There is growing evidence that PGPR release a variety of metabolites to promote plant growth and reduce the negative effects of salt stress (Abbas et al., 2019; Lucke et al., 2020a). This study evaluated the effect of inoculation with ZS-3 on the growth and development of plants under 100 mM NaCl stress. The growth of *A*. *thaliana* was significantly inhibited by salt stress, but inoculation with ZS-3 reduced the negative effects and significantly improved growth.

The dynamic modulation of root structure (RSA) can be considered one of the most important defense mechanisms for roots to resist stress (Postma and Lynch, 2011). A deep and robust root system facilitates plant uptake of nutrients from the soil (Zhou et al., 2016). In this study, plants inoculated with ZS-3 showed no significant promotion of primary root growth but had more lateral root formation than control plants. This is typically associated with growth hormone accumulation in plant roots (Wakeel et al., 2018; Wang et al., 2021). *Bacillus megaterium* WW1211 has been shown to inhibit the growth of primary roots by modification of the auxin cycle and auxin redistribution in the root meristem (Wang et al., 2021). However, the mechanism of growth promotion is different among different isolates of *B*. *megaterium* (Wang et al., 2021). The possibility that polyamines improve the RSA of plants cannot be ruled out. Polyamines control cell division and differentiation in plants (Wu et al., 2012), playing an important role in primary root, lateral root and adventitious root development (Wu et al., 2012). Plants inoculated with Spd-producing BOFC15 had longer primary roots and more lateral roots (Zhou et al., 2016). In contrast to BOFC15, ZS-3 not only produces spermidine but also produces putrescine (unpublished). The altered root architecture may be attributed to the comediation of multiple metabolites by ZS-3.

Chlorophyll content is a key predictor of both plant health and photosynthetic tolerance to NaCl stress (Li et al., 2010; Heidari, 2011). The chlorophyll content in the leaves of *A*. *thaliana* was reduced under salt stress. The color of plant leaves was darker under the treatment with strain ZS-3, together with increased photosynthesis in the plants. This is directly related to the increase in chlorophyll content. Chlorophyll, which is usually responsible for green color in plants, is an important pigment for converting light into chemical energy (Kang et al., 2019). In transgenic lines with dark green leaves, the chlorophyll content was significantly higher than that in the wild type (Yang et al., 2011). In addition, other possible reasons for the darkening of leaf color were induction by the VOCs of ZS-3. Compared with the control, the ZS-3-treated leaves all showed a darker green color in the presence and absence of salt stress (Supplementary Figure S1A).

The accumulation of osmotic regulators maintains intracellular osmotic pressure homeostasis, which is one of the important mechanisms of plant tolerance to salt stress (Abbas et al., 2019). Soluble sugars are one of the key substances in plant osmoregulation (Zang et al., 2019). The results showed that ZS-3 promoted the accumulation of soluble sugars in plants under salt stress. Many studies have confirmed that PGPR promote the accumulation of soluble sugars and proline in plants and maintain the water status of cells to resist salinity-induced osmotic stress. For example, inoculation with *Kocuria rhizophila* Y1 increased the soluble sugar and proline contents of maize under NaCl stress (Li et al., 2020a). However, inoculation with ZS-3 significantly reduced the plant proline content. This is consistent with the results of studies such as Hmaeid et al. (2019) and Liu et al. (2020), where bacterial inoculation significantly reduced the proline content of plants under salt stress conditions. Plants inoculated with PGPR were not exposed to high salt stress and therefore accumulated less proline in the presence of PGPR (Shukla et al., 2012; Liu et al., 2020).

The control of Na ^+^ homeostasis is essential for maintaining normal plant growth under salt stress (Jiang et al., 2019). ZS-3 increases the K^+^/Na^+^ ratio by directly limiting the accumulation of Na^+^, rather than by increasing the K^+^ content, in the presence or absence of salt stress. This is closely related to the regulation of ion transport proteins by ZS-3. PGPR downregulate *HKT1* expression and limit Na^+^ uptake by roots, thereby improving plant salt tolerance (Pinedo et al., 2015; Liu et al., 2020). In addition, segregation of Na^+^ from the cytoplasm to vesicles is one of the key strategies for mitigating Na^+^ toxicity in plant cells. *AVP1* encodes a vacuolar H^+^-pyrophosphatase that pumps H^+^ into vesicles, which acidify to generate an H^+^ gradient across the membrane. *NHX1* pumps Na^+^ into the vesicle compartment to exploit the potential difference in H^+^, thus effectively reducing the toxic effect of Na^+^ (Rivandi et al., 2011). ZS-3 regulates *HKT1*, *NHX1*, and *AVP1*, so we speculate that ZS-3 restricts Na^+^ uptake and sequesters Na^+^ in plant vesicles, which is one of the effective strategies by which ZS-3 alleviates salt stress in plants. Another possible strategy is that ZS-3 phagocytoses Na^+^ from the environment. A general consensus is that high external Na^+^ concentrations interfere with the ability of plants to access K^+^ due to competition between Na^+^ and K^+^ for the major binding sites of K^+^ channels or transport proteins (Wang et al., 2020). This is one of the pathways by which PGPR alleviate ionic stress in plants by reducing plant Na^+^ accumulation or increasing K^+^ uptake, thereby increasing the K^+^/Na^+^ ratio (Gong et al., 2020). However, plants inoculated with ZS-3 showed less Na^+^ than uninoculated plants in the absence of salt stress. This indicates that ZS-3 has an additional mechanism to limit plant Na^+^ accumulation. The concentrations of intracellular and extracellular Na^+^ were lower in *Rahnella aquatilis* JZ-GX1 inoculated with LB liquid medium (CK) under salt stress, indicating that the bacteria can consume Na^+^ in the medium (Li et al., 2021). ZS-3 is a salt-tolerant strain that tolerates 8% salt in LB, corresponding to moderate salt stress (Lu et al., 2018). The salt tolerance mechanism of the ZS-3 strain may also play an important role in the process of interaction with plants.

Antioxidant enzyme systems and non-enzymatic antioxidants are important defense systems that effectively mitigate the oxidative stress of plants under environmental stress (Gill and Tuteja, 2010). ZS-3 increased the contents of POD, CAT, and GSH under salt stress. POD and CAT reduce H_2_O_2_ to H_2_O and O_2_ (Nie et al., 2019). GSH is a potential quencher of ROS in organisms (Zheng et al., 2020). Interestingly, ZS-3 did not reduce the MDA and H_2_O_2_ contents of plants but instead promoted the accumulation of MDA and H_2_O_2_. Although the accumulation of ROS affects many cellular functions, it is noteworthy that whether ROS exert destructive, protective or signaling effects depends on the delicate balance of ROS production and scavenging at the right site and time (Gill and Tuteja, 2010). Since the plants showed excellent resistance to salt stress by interacting with ZS-3, we hypothesized that the accumulation of ROS might have been due to the excessive accumulation of bacteria and their metabolites in the medium at the late growth stages. Consistent with our speculation, there was no excessive accumulation of ROS in plants treated with VOC of ZS-3 (Supplementary Figure S1B). The high concentration of nutrients caused an additional burden on the plants, which could be one of the reasons for the excessive accumulation of ROS. The regulation of salt tolerance in plants by ZS-3 may not depend on the mechanism of action of antioxidant enzymes to scavenge ROS.

Three plant hormones, SA, JA, and ET, regulate plant responses to a range of biotic and abiotic stresses, as well as plant growth and development (Dar et al., 2015; Niu et al., 2016). Transcriptomics has shown that PGPR mediate plant tolerance to salt stress through activation of the JA/ET signaling pathway (Liu et al., 2017). Moreover, the transcriptome data show that all 20 genes encoding PR proteins were upregulated under 100 mM NaCl stress (Liu et al., 2017). Consistent with our results, the three defense signaling pathways of JA/ET and SA in Arabidopsis were activated by ZS-3. The results indicate that IST of Arabidopsis may be activated by ZS-3 in response to salt stress.

In addition, several studies have shown that PGPR alter the epigenetic characteristics of host plants to provide beneficial selectable variation and regulate crosstalk between phytohormones (Tiwari et al., 2021). Evidence supports the coordinated role of the Arabidopsis transcription factor *ERF1* in integrating JA and ET signaling (Dimopoulou et al., 2019). We found that the JA signaling pathway was first regulated by ZS-3 (3d). ZS-3 then integrated the JA/ET signaling pathway by upregulating *ERF1* expression (5 days; Lucke et al., 2020b). At the same time, the transcript levels of *PDF1.2* were upregulated (5 days). JA/ET signaling induces *PDF1.2*, and SA signaling upregulates *PR1*; these two processes show an antagonistic relationship when responding to biotic stresses (Sooklal et al., 2020). However, it has been reported that *NPR1* has a role in fine-tuning the antagonistic regulation of SA and JA/ET signaling to control gene expression (Zhang et al., 2019). Our results indicate that *PR1* gene (8 days) expression is upregulated following activation of the *NPR1* gene (5d) by ZS-3. In other words, the SA signaling pathway in *A*. *thaliana* is well-prepared to cope with salt stress. However, *PR1* was downregulated in the early stage (3 days). This implies that SA may be antagonized by JA/ET in early signaling crosstalk but undergoes dynamic changes at a later stage. What activates their coordinated action needs to be further investigated. Crosstalk between plant hormones can reprogram genetic mechanisms to influence defense responses and minimize stress effects, thereby improving stress tolerance in plants (Per et al., 2018). Although the physiological and molecular mechanisms of PGPR-plant interactions have been reported in many studies, little is known about the interaction of proteins and small molecules from beneficial microorganisms with different branches of the plant immune system (Lucke et al., 2020b). Further insights are needed in the future to reveal which metabolites trigger plant defense responses. A large number of metabolites associated with different plant hormones play a role in plant defense and plant-environment interactions (Lucke et al., 2020b). In addition, further studies are needed to evaluate the effectiveness of ZS-3 in mitigating salt stress in crops under field conditions.

## Conclusion

**Figure 7:**
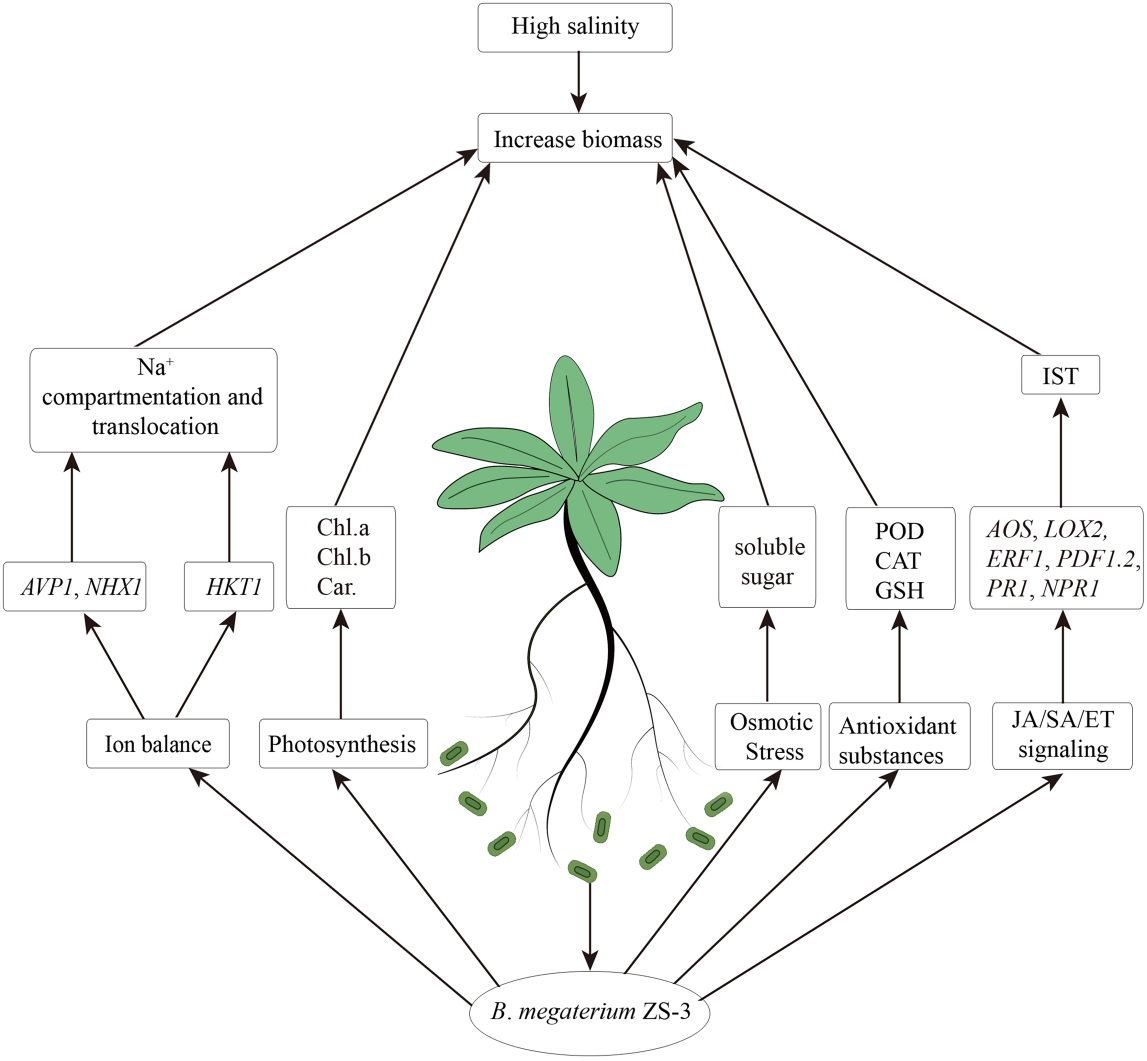
Possible mechanisms by which *Bacillus megaterium* strain ZS-3 mediates salt stress tolerance in *Arabidopsis thaliana* under salt stress.

In this study, ZS-3 was found to promote plant growth under salt stress. The improvement in the growth of *A*. *thaliana* induced by ZS-3 can be attributed to the integration of multiple physiological processes, including regulation of photosynthetic pigment synthesis (chlorophyll and carotenoids), accumulation of osmotic regulators (soluble sugars), increases in antioxidant substances (CAT, POD, GSH), or activation of ion transporter gene expression (*NHX1*, *AVP1*, and *HKT1*), which led to an increased ratio of K^+^/Na^+^ (Figure 7). In addition, the transcript levels of defense response genes related to the SA and JA/ET synthesis pathways (*NPR1*, *PR1*, *ERF1*, *PDF1.2, LOX2*, and *AOS*) were differentially activated, which helped to improve the salt tolerance of *A*. *thaliana*. In summary, this report shows that ZS-3 mitigates the negative effects of salt stress on plants. We demonstrate that inoculation with ZS-3 is a valuable and eco-friendly biotechnological approach to improve agricultural practices and production under saline conditions. The results of this study provide new insights into the induction of salinity tolerance by PGPR, and in the next step of our research, we will determine which substances of ZS-3 induce systemic salinity tolerance and activate systemic tolerance in plants.

## Data Availability Statement

The original contributions presented in the study are included in the article/Supplementary Material, and further inquiries can be directed to the corresponding author.

## Author Contributions

L-NS and J-RY conceived and designed the experiments. L-XL provided valuable advice on data analysis and the manuscript. H-MS assisted with the statistical analysis. L-NS analyzed the data and drafted the manuscript. J-RY revised the manuscript. All authors contributed to the article and approved the submitted version.

## Funding

This work was financially supported by the Science and Technology Project of Shanghai Greening Bureau (no. G191208), the Chinese State Forestry Administration Special Research Program for Forestry Sectors Beneficial to Public (no. 201304404), and the Priority Academic Program Development of Jiangsu Higher Education Institutions (PAPD).

## Conflict of Interest

The authors declare that the research was conducted in the absence of any commercial or financial relationships that could be construed as a potential conflict of interest.

## Publisher’s Note

All claims expressed in this article are solely those of the authors and do not necessarily represent those of their affiliated organizations, or those of the publisher, the editors and the reviewers. Any product that may be evaluated in this article, or claim that may be made by its manufacturer, is not guaranteed or endorsed by the publisher.

## Supplementary Material

Supplementary Figure S1 Effect of volatiles gases (VOC) from ZS-3 on phenotypic **(A)** and MDA content **(B)** in *A*. *thaliana*. Six-day-old seedlings were transplanted and cultured in dichotomous dishes for 14 days. VOC from ZS-3 exhibited a significant pro-growth effect on plants and induced dark green leaves in the presence or absence of salt stress. The MDA content of *A*. *thaliana* was measured at 14 d. Control: PBS inoculation only, ZS-3: bacterial inoculation, Voc-ZS-3: VOCs inoculation. The results are the mean ± standard deviation of three independent experiments. Different lowercase letters on the bars represent significant differences between treatments, based on one-way ANOVA (Duncan’s multiple range, *p* < 0.05).
