# Supplementary material for: The Endophytic Strain ZS-3 Enhances Salt Tolerance in Arabidopsis thaliana by Regulating Photosynthesis, Osmotic Stress, and Ion Homeostasis and Inducing Systemic Tolerance
Source: Front Plant Sci. 2022 Mar 21;13:820837. doi: 10.3389/fpls.2022.820837 (PMC8977589; doi:10.3389/fpls.2022.820837)
Supplement: Supplementary file 1 [file Table_1.DOCX]

Supplementary Material

Supplementary Table 1 qRT-PCR primer sequences used in this study.

| Name | Sequence | Target gene |  |
| --- | --- | --- | --- |
| Actin2-F | CCTGCCATGTATGTTGCCATT | Internal reference | (Wu et al. 2018) |
| Actin2-R | AATCGAGCACAATACCGGTTGT |  |  |
| NHX1-F | GGATGCCTTGGACATTGAC | sodium hydrogen exchanger 1 | (Liu et al. 2020) |
| NHX1-R | GAAAGACGAACGCTGCTCT |  |  |
| AVP1-F | GTGGGATCTACACTAAGGCTG | proton-pyrophosphatase *AVP1* | (Jiang et al. 2017) |
| AVP1-R | TCCCATACCAGCAATGTCAC |  |  |
| HKT1-F | GGTCTCATCTGGCTCCTAATC | high-affinity K^+^ transporter 1 | (Liu et al. 2020) |
| HKT1-R | ATGTAACCATACTCGTCACGC |  |  |
| PR1-F | AGGTGCTCTTGTTCTTCCCT | Detection of expression of *PR1* | (Wu et al. 2018) |
| PR1-R | ACCCCAGGCTAAGTTTTCCC |  |  |
| NPR1-F | ACCGATAACACCGACTCCTC | Detection of expression of *NPR1* | (Wu et al. 2018) |
| NPR1-R | GCACCGGTGGAAAGAAACTT |  |  |
| AOS-F | TGAGTTTGTGCCGGAGAGAT | Detection of expression of *AOS* | (Wu et al. 2018) |
| AOS-R | ATCACAAACAACCTCGCCAC |  |  |
| ERF1-F | AGGATGGTTGTTCTCCGGTT | Detection of expression of *ERF1* | (Wu et al. 2018) |
| ERF1-R | AGACCCCAAAAGCTCCTCAA |  |  |
| PDF1.2-F | CACCCTTATCTTCGCTGCTC | Detection of expression of *PDF1,2* | (Wu et al. 2018) |
| PDF1.2-R | GCACAACTTCTGTGCTTCCA |  |  |
| LOX2-F | ATCAACGCTCGTGCACGCCA | lipoxygenase 2 | (Chu et al. 2019) |
| LOX2-R | CCGCGGGTAAGCCTTCCTGG |  |  |

Chu, T N, Tran, B T H, Van Bui, L and Hoang, M T T, 2019. Plant growth-promoting rhizobacterium pseudomonas ps01 induces salt tolerance in arabidopsis thaliana. *BMC Research Notes*. 12:11.10.1186/s13104-019-4046-1

Jiang, W, Sun, L, Yang, X, Wang, M, Esmaeili, N, Pehlivan, N, Zhao, R, Zhang, H and Zhao, Y, 2017. The effects of transcription directions of transgenes and the gypsy insulators on the transcript levels of transgenes in transgenic arabidopsis. *Scientific Reports*. 7:14757.10.1038/s41598-017-15284-x

Liu, S, Tian, Y, Jia, M, Lu, X, Yue, L, Zhao, X, Jin, W, Wang, Y, Zhang, Y, Xie, Z and Wang, R, 2020. Induction of salt tolerance in arabidopsis thaliana by volatiles from bacillus amyloliquefaciens fzb42 via the jasmonic acid signaling pathway. *Frontiers in Microbiology*. 11:10.3389/fmicb.2020.562934

Wu, G, Liu, Y, Xu, Y, Zhang, G, Shen, Q and Zhang, R, 2018. Exploring elicitors of the beneficial rhizobacterium bacillus amyloliquefaciens sqr9 to induce plant systemic resistance and their interactions with plant signaling pathways. *Molecular Plant-Microbe Interactions®*. 31:560-567.10.1094/mpmi-11-17-0273-r
