# Supplementary figures and images for: The Endophytic Strain ZS-3 Enhances Salt Tolerance in Arabidopsis thaliana by Regulating Photosynthesis, Osmotic Stress, and Ion Homeostasis and Inducing Systemic Tolerance
Source: Front Plant Sci. 2022 Mar 21;13:820837. doi: 10.3389/fpls.2022.820837 (PMC8977589; doi:10.3389/fpls.2022.820837)

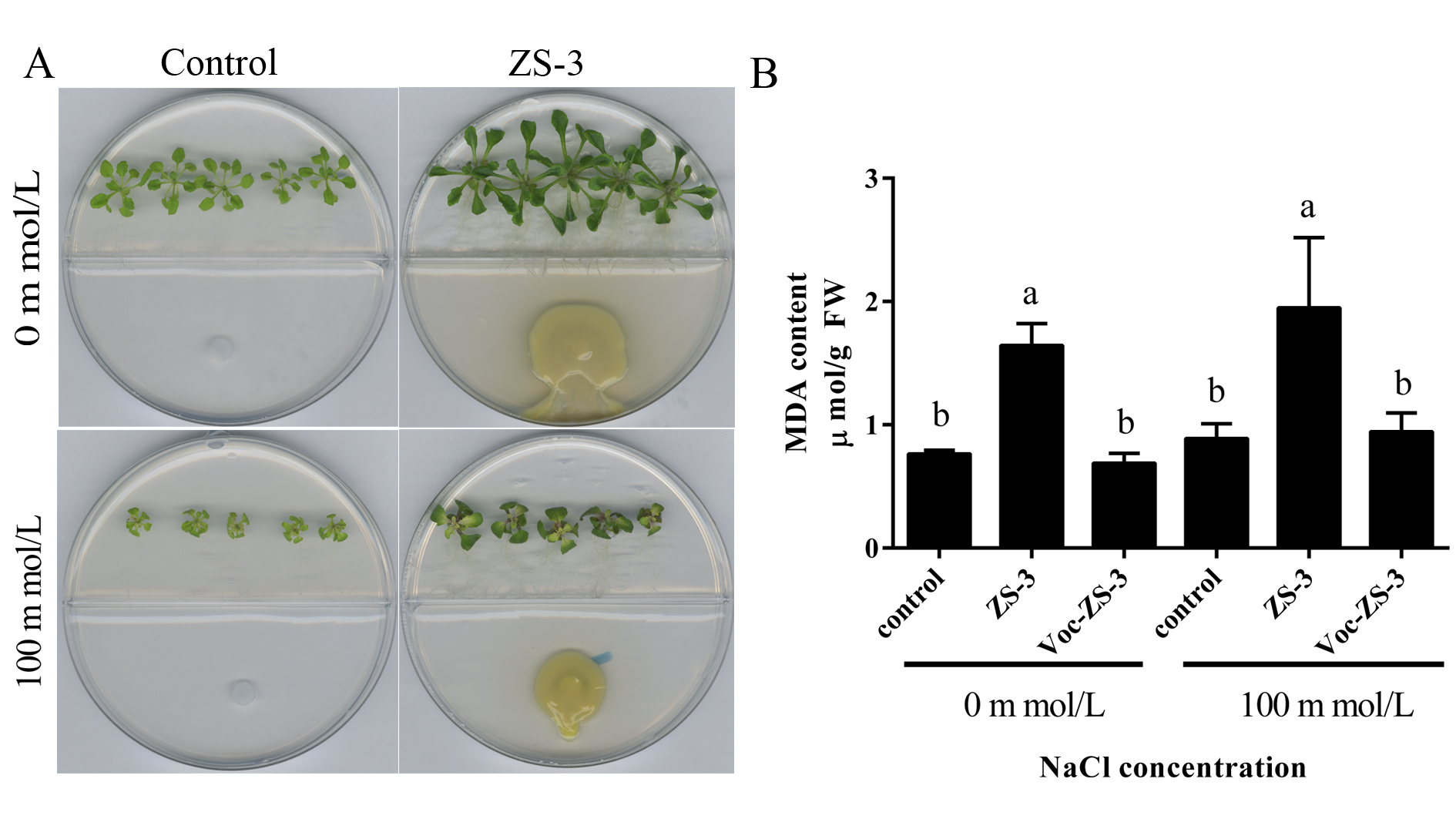

Supplement: Supplementary Figure S1 — Effect of volatiles gases (VOC) from ZS-3 on phenotypic (A) and MDA content (B) in A. thaliana. Six-day-old seedlings were transplanted and cultured in dichotomous dishes for 14 days. VOC from ZS-3 exhibited a significant pro-growth effect on plants and induced dark green leaves in the presence or absence of salt stress. The MDA content of A. thaliana was measured at 14 d. Control: PBS inoculation only, ZS-3: bacterial inoculation, Voc-ZS-3: VOCs inoculation. The results are the mean ± standard deviation of three independent experiments. Different lowercase letters on the bars represent significant differences between treatments, based on one-way ANOVA (Duncan’s multiple range, p < 0.05). [file Image_1.tif]
